# Interaction of Protein Preloads and Physical Activity on Intake of an Ultra-Processed, High Sugar/High Fat Food/Low Protein Food

**DOI:** 10.3390/nu14040884

**Published:** 2022-02-19

**Authors:** Jennifer A. Nasser, Eram Albajri, Lisa Lanza, Abigail Gilman, Mansour Altayyar, Dimitra Thomopoulos, Michael Bruneau

**Affiliations:** 1 Department of Nutrition Sciences, Drexel University, Philadelphia, PA 19102, USA; lisalanza17@yahoo.com (L.L.); aed@gmail.com (A.G.); maa449@drexel.edu (M.A.); dnt38@drexel.edu (D.T.); 2 Clinical Nutrition Department, Faculty of Applied Medical Sciences, King Abdulaziz University, Jeddah 21589, Saudi Arabia; dr.eram@hotmail.comKing; 3 Department of Health Sciences, Drexel University, Philadelphia, PA 19102, USA; mlb425@drexel.edu

**Keywords:** loss of control eating, hedonic hunger, protein preload, ultra-processed food

## Abstract

“Loss of control, LOC” eating is a major contributor to the development of obesity. Dietary protein is known to promote satiety, but little attention has been paid to the ability of protein, consumed in close proximity to snacking (20 min), to reduce the intake of ultra-processed, low-protein snack foods. We hypothesized that a high-protein preload (HP, 8 g of protein) consumed in close proximity to eating an ultra-processed snack food would reduce intake of the snack food as compared to a low-protein preload (LP, 1.2 g of protein). Two laboratory test meals were conducted, and the intake of ice cream (1.99 kcal/gram) after consuming dairy-based liquid preloads was measured. Habitual physical activity, a potential modulator of satiety, was assessed by a self-reporting questionnaire. Thirty (responders) out of 50 participants reduced their intake of ice cream after the HP preload, with a significant difference in intake observed between the responders and non-responders (−30 ± 25 and 18 ± 18 g, F (1, 49) = 54.36, *p* < 0.001 for responders and non-responders, respectively). Our data demonstrate that protein consumed in close proximity to ultra-processed snack food can reduce caloric intake by ~60 kcal, which could potentially reduce body weight by at least 5 pounds per year.

## 1. Introduction

A major contributor to the development of obesity, a current public health problem in the US, is eating when not physically hungry or “loss of control, LOC” eating [1]. Frank et al. [2] demonstrated that LOC eating also has a negative impact on successful weight loss and maintenance of the “resulting reduced weight state”. Dietary protein is known to promote the most satiety—that is, the longest inter-meal interval—but little attention has been paid to the ability of protein to reduce or curtail acute LOC eating of ultra-processed food, recently identified as a driver in the development of obesity [3]. 

Dunford and Popkin [4] reported that intra-meal eating or “snacking” averages about 500 kcal/day (~25% of the recommended daily energy intake), making it a major contributor to energy balance. LOC eating is based on a conditioned response to palatability, stress, or environmental cues—in effect, “hedonic” hunger, as opposed to physiological hunger based on nutrient depletion. Ultra-processed foods are purposefully designed to be hyperpalatable, thus appealing to “hedonic” hunger [5]. The food intake during an episode of LOC eating can range anywhere from 600 to 3000 kcals [6,7,8,9,10] and is usually lower in % protein calories and higher in % fat and sugar calories than controlled eating episodes [10]. De Carvalho et al. [11] found that high-protein diets were associated with enhanced sensations of fullness and satiety in overweight and obese individuals. The efficacy of protein to dampen “hedonic” hunger and curtail LOC during an eating episode was suggested by Latner et al. [12], but the current treatment for loss of control eating does not focus on the ability of dietary protein to limit the intake of other macronutrients. High-protein snacks improve appetite control, satiety, and reduce the subsequent food intake in healthy weight individuals [13,14]. However, the ability of protein consumed proximal to (20 min before) the intake of high-sugar, high-fat, low-protein (HSFLP) ultra-processed food to limit the LOC of HSFLP has received little attention. This timing scheme fits easily into common meal and snacking episodes and, if shown to be effective at reducing the HSFLP intake, would provide a useful, consumer-friendly intervention to assist in body weight control.

To address the question of whether protein can reduce acute LOC eating, we conducted a study where participants consumed two preloads closely matched for energy content but varying in protein content (1.2 g versus 8 g) on the subsequent intake of a common ultra-processed food high in sugar and fat and low in protein (HSFLP): vanilla ice cream. Since our study was conducted in “free-living” individuals, it was necessary to consider another potential modulator of appetite, namely habitual physical activity. Acute decreases in appetite and short-term increases in energy expenditure are associated with bouts of physical activity independent of the macronutrient content of a meal, although the magnitude of such changes varies between individuals [15]. Habitual physical activity also tends to dampen food reward signaling [16,17], but the ability of habitual physical activity to modulate the effect of protein intake in close proximity to reduce the intake of energy-dense ultra-processed foods is still in question. We assessed habitual physical activity and habitual eating behavior by self-reporting questionnaires and measured the response of regions of the prefrontal cortex during eating using functional near infrared spectroscopy (fNIR). Data on the interaction of habitual physical activity and food intake following the consumption of protein preloads will be presented in this manuscript. Data from fNIR measurements during eating, as well as the interaction of self-reported habitual eating behavior with fNIR, will be presented elsewhere. We hypothesized (a) that the intake of HSFLP ultra-processed food would be less after consuming the high-protein preload and (b) that the intake of HSFLP after the high-protein preload would correlate negatively with habitual physical activity.

## 2. Materials and Methods

This study was conducted with oversight from the Drexel University Institutional Review Board, and all participants provided written informed consent prior to taking part in any experimental activities.

### 2.1. Participants

Fifty healthy participants (Table 1) (25 M, 25 W) completed two test sessions in this study. Participants reported being weight stable for the preceding 3 months, between the ages of 18 and 55 years, with a body mass index (kg/m^2^, BMI) 20–40 that was calculated from their measured weight and height. The exclusion criteria included a current diagnosis of a metabolic disorder (diabetes, thyroid illness, or renal disease) or cancer; a past or current diagnosis of eating disorders or neurological disease; the current use of antidepressants and/or antipsychotic medication; pregnancy or nursing within the past 12 months; and an allergy to wheat, soy, tree nuts, corn, or tomato products. Additionally, individuals reporting that they never eat ice cream or were allergic to, or intolerant of, dairy foods were also excluded from the study.

Participants were recruited through flyers placed on Drexel University campuses and in the surrounding Philadelphia Metro Area. The screening of participants and scheduling of sessions was done by phone, with participants self-identifying an interest in the study by contacting our laboratory. During the scheduling, women participants were instructed to track their menstrual cycle, which might serve as a potential covariate for data analysis.

### 2.2. Experimental Procedures

Participants attended two test sessions, each held on a different day and separated by at least 2 days from each other. The test sessions were conducted between 12:30 and 4:30 p.m. Since participants were “free-living”, both sessions were held at the same time of day (±30 min) per person as a means of standardizing the pretesting conditions. Participants were instructed to have a light meal, then abstain from eating anything or consuming caloric beverages, coffee, or tea for three hours prior to the scheduled session. During the first test session, participants had weight, height, waist, and head circumference measured, as well as consumed one of the protein preloads. They rated their hunger on a 100-mm visual analog scale (VAS, anchored at 0 with Not Hungry and at 100 with Extremely Hungry) before and 15 min after consuming a preload. After consuming the preload, participants rested for 15 min, rated their hunger again on the VAS, then were fitted with the fNIR wearable sensor. A two-minute fNIR recording was obtained prior to participants being provided with the HSFLP (a name brand vanilla ice cream, (1.99 kcal/gram) at 20 min post-preload and allowed to eat ad libitum for a maximum of 10 min. (Note: We chose a 10-min eating period based on data from a prior experiment in our lab [18] where individuals who had not consumed any preloads ate ice cream ad libitum for a maximum of 10 min). The presentation of the preloads was randomized between participants. The preloads consisted of dairy protein-based beverages with chocolate flavoring, provided as 8 ounces, with the LP preload delivering 1.2 g of protein, 111 kcal and the HP preload delivering 8 g of protein, 90 kcal. Participants were not informed of the protein content of the preloads. 

During the second session, participants again rated their hunger (before and after consuming the preload) using the VAS and completed the Three Factor Eating Questionnaire [19], the Power of Food Scale [20], and a physical activity questionnaire [21] at the end of the session after completing the ad libitum food consumption. The physical activity questionnaire (PAQ-M) cited was chosen because it was designed to capture the physical activity of daily living activities, as well as planned recreational exercise activities, and had been validated against the often-used Paffenbarger (PAQ-P) [22] Participants registered their hourly activity using 8 response categories for the 12-item questionnaire. The average hours/week of activity were calculated per Rubenstein [21] using “the midpoints of the eight response categories, i.e., 0 (none), 0.5 (<1), 1.5 (1–2), 4 (3–5), 7.5 (6–9), 14.5 (10–19), 24.5 (20–29), and 35 (≥35)”.

## 3. Results

### 3.1. Protein Content of Preload and Intake of HSFLP

In Table 2, the reported hunger ratings are those recorded at 15 min after consuming the preloads before the ad libitum intake. The average hunger ratings under both preload conditions was 40, suggesting that participants were “not physically hungry” prior to ad libitum consumption of the HSFLP. Table 2 also shows a small but significant reduction in ad libitum HSFLP intake that was observed after consumption of the high-protein preload (*p* = 0.025). Additionally, the intake after the HP preload, but not the LP preload, was significantly, and negatively, correlated with habitual physical activity (r = −0.309, *p* = 0.029 and r =−0.26, *p* = 0.068 for HP and LP, respectively). This finding agrees with our stated hypothesis.

#### Post-Hoc Analysis

A frequency distribution of the difference in intake between the two preloads (HP and LP) showed that 30 out of 50 participants consumed less HSFLP after the HP preload compared to the LP preload. This response of 60% of our participants is in agreement with our hypothesis that a high-protein preload can reduce “eating when not physically hungry” or “LOC” eating in individuals who were not physically hungry based on their hunger rating of 40 out of 100. To determine how much of a reduction in intake occurred between those who responded to the HP preload and those who did not, we created two groups based on whether the difference in HSFLP intake between the two preloads was negative (i.e., the responders) or positive and/or zero (i.e., the non-responders). The difference in ad libitum intake (in grams) between the responders and non-responders was significant (−30 ± 25 and 18 ± 18 g, F (1, 49) = 54.36, *p* < 0.001 for responders and non-responders, respectively). This difference amounted to an average reduction in the responders of 60 kcals (based on 1.99 kcal/gram energy density of the HSFLP) after the HP preload. There were no significant differences in any of the physiological variables assessed (BMI, age, and waist circumference) or level of weekly physical activity between the responder and non-responder groups (*p* > 0.05).

### 3.2. Influence of Habitual Physical Activity on Protein-Induced Satiation

Table 3 shows that physical activity and ad libitum intake of ultra-processed foods were negatively correlated in those who were not overall responders to protein-induced acute satiety (r = −0.498, *p* = 0.03 and r = −0.56, *p* = 0.13 for LP versus HP, respectively). There was no difference in effect seen in the non-responders between preloads and no effect of physical activity seen in those who responded to protein-induced acute satiety. 

## 4. Discussion

We assessed the ability of protein consumed in close proximity (20 min) to an ultra-processed snack to reduce LOC eating of the ultra-processed snack. Additionally, we examined the interaction of habitual physical activity to modulate a protein-satiating effect. Our data demonstrated that 60% of participants mounted a response to a high-protein preload with an acute decrease in intake of an HSFLP ultra-processed food, (vanilla ice cream). We also showed that, among those responsive to the high-protein preload, no additional reduction in HSFLP intake could be ascribed to hours per week of habitual physical activity. Additional analysis of a measure of exercise intensity provided by the PAQ-M (METS, kcal/kg/week) showed no statistical difference between responders and non-responders, providing further evidence of no modulating effect of habitual physical activity on protein-associated reduction in the HSFLP intake. However, among those non-responsive to the high-protein preload, habitual physical activity was negatively correlated with the intake of HSFLP. It was not evident why 40% of our participants were non-responders to the high-protein preload, especially given that there were no significant differences observed in the physiological variables between the responders and non-responders. The absence of a difference in intake between the LP and HP preloads in the “non-responders” suggests that habitual physical activity can have an overall dampening effect on “eating when not physically hungry” through the better coupling of appetite and satiety. This would be consistent with the results seen in those with BMI ≥ 27, as reviewed by Dorling et al. [15]. 

This study examined the effect of protein consumption in close proximity to acute LOC of ultra-processed snack foods, namely 20 min after preload consumption. Most studies examining protein-satiating/satiety effects use a 1–4-h time period between protein intake and subsequent food intake [23,24,25,26,27]. Our use of a 20-min period places our results in the realm of techniques for controlling not only “snacking” but also “premeal” intakes, such as appetizers at formal lunches and dinners or responses to a child’s “I’m hungry” before the formal meal time. The 30-g reduction in HSFLP amounts to a 60-kcal reduction in intake, which has real-world clinical implications for body weight control. Applying this approach to even one snack or meal per day for a week would result in a decrease of at least 420 kcals per week. That level of caloric reduction coupled with an additional 23% thermic effect of protein [28] could lead to at least a 5-pound weight loss in one year or contribute to the energy balance needed to maintain a healthy weight. More studies on the intake of protein in close proximity to further food intake, as well as any contribution of habitual physical activity, which appears to act independently from protein in promoting the reduced intake of an ultra-processed snack, seem warranted.

## 5. Conclusions

Protein is known to reduce appetite and increase satiety [13,14]. In this study we demonstrate that providing 8 grams of protein, the amount contained in a glass of milk for example, in close proximity (20 min) to consuming an energy dense ultra-processed food and reduce gram and caloric intake of that food. This finding places protein dosing “in close proximity to further eating” in the realm of a practical approach to reducing intake of ultra-processed food, a major contributor to development of obesity [3].

## Figures and Tables

**Table 1:** Demographics.

Variable	Sample *n* = 50
Body Mass Index (BMI, kg/m^2^)	27.1 ± 3.9
Age	27.1 ± 3.9
Physical Activity (hours/week)	49 ± 22

**Table 2:** Intake of high-sugar/fat low-protein food after preloads varying in protein contents.

Participants	Hunger (Low-Protein)	Hunger (High-Protein)	Low-Protein Preload	High-Protein Preload
All *n* = 50	40 ± 24	41 ± 27	115 ± 60 g *	105 ± 62 g *

LP = low-protein preload, HP = high-protein preload. Comparison of HSFLP intake after LP and HP preloads by paired *t*-test: *t* (49) = 2.3, * *p* = 0.025.

**Table 3:** Correlation of high-sugar/fat low-protein intake and physical activity by the responder group.

Responder Group	Low-Protein Preload	High-Protein Preload
Non-responder	r = −0.498, *p* = 0.03	r = −0.56, *p* = 0.013
Responder	r = −0.05, *p* = 0.79	r = −0.03, *p* = 0.88

## Data Availability

The data will be made available through deposition in a public data site after publication of the accompanying fNIR data.
